# Systemic Immune-Inflammatory Biomarkers in Epithelial Ovarian Cancer: Subgroup-Dependent Prognostic Performance and Integrated Risk Stratification

**DOI:** 10.3390/ijms27177777

**Published:** 2026-08-30

**Authors:** E Sun Paik, Young Eun Chung, Seoyoung Youn, Seongyun Lim, Jun-Hyeong Seo, Chel Hun Choi, Tae-Joong Kim, Jeong-Won Lee, Yoo-Young Lee

**Affiliations:** 1 Department of Obstetrics and Gynecology, Kangbuk Samsung Hospital, Sungkyunkwan University School of Medicine, Seoul 03181, Republic of Korea; 2 Gynecologic Cancer Center, Department of Obstetrics and Gynecology, Samsung Medical Center, Sungkyunkwan University School of Medicine, 81 Irwon-ro, Gangnam-gu, Seoul 06351, Republic of Korea

**Keywords:** epithelial ovarian cancer, tumor microenvironment, systemic immune-inflammation index, prognostic biomarker, interleukin-6, neutrophil-to-lymphocyte ratio, platelet-to-lymphocyte ratio, inflammation

## Abstract

Systemic immune-inflammation index (SII), neutrophil-to-lymphocyte ratio (NLR), and platelet-to-lymphocyte ratio (PLR) are surrogate markers of the systemic immune-inflammatory response proposed as perioperative prognostic biomarkers in epithelial ovarian cancer (EOC), yet their performance across subgroups remains unexamined. In 373 EOC patients undergoing primary cytoreductive surgery, postoperative day-1 changes (ΔSII, ΔNLR, ΔPLR) were compared for overall survival (OS) and progression-free survival (PFS) across 10 subgroups, and a Clinical-Inflammatory Risk Score (CIRS) combining inflammation with stage and residual disease was developed. Individual markers showed modest discrimination (area under the curve [AUC] 0.579–0.615); the marker with the highest AUC varied by context, with ΔPLR ranking first most often, notably in non-serous tumors, though none was statistically superior. Seeking a molecular counterpart, two public transcriptomic cohorts were re-analyzed: VWF was consistently higher in clear cell than serous carcinoma, whereas IL6–STAT3-related differences were cohort-dependent. The CIRS achieved AUCs of 0.752 (OS) and 0.764 (PFS), a six-fold mortality gradient (7.2% vs. 44.4%), and remained independently associated with OS and PFS (hazard ratio 2.55 and 2.24 per standard deviation), though discrimination was not significantly better than stage and residual disease alone. These biomarkers show subgroup-dependent prognostic value, and the CIRS provides an exploratory risk-stratification framework warranting prospective validation.

## 1. Introduction

Epithelial ovarian cancer (EOC) remains the most lethal gynecologic malignancy, accounting for approximately 207,000 deaths annually worldwide [1,2]. Despite advances in surgical technique and the introduction of PARP inhibitors as maintenance therapy, the five-year overall survival (OS) for advanced-stage disease remains below 30% [1,3]. Accurate postoperative risk stratification is essential for individualizing surveillance intensity, guiding clinical trial enrollment, and informing maintenance therapy decisions [3].

Cytoreductive surgery is the cornerstone of EOC management, but the surgical procedure itself triggers a profound perioperative inflammatory response that may paradoxically promote tumor recurrence. The extensive tissue dissection required for radical cytoreduction activates the hypothalamic–pituitary–adrenal axis, releases catecholamines, and induces marked shifts in circulating immune cell populations [4,5]. Postoperative immunosuppression—involving natural killer cell dysfunction, myeloid-derived suppressor cell expansion, and relative lymphopenia—creates a permissive window for residual tumor cell proliferation and awakening of dormant micrometastases [6,7]. Praiss et al. recently demonstrated that plasma interleukin-6 rises substantially during cytoreductive surgery for high-grade serous EOC and activates pro-tumorigenic inflammatory signaling pathways, providing biological rationale for monitoring the perioperative inflammatory response [8]. At the molecular level, this cytokine surge propagates a self-reinforcing cascade: IL-6 promotes hepatic thrombopoietin release and reactive thrombocytosis, and the activated platelets in turn shield circulating tumor cells and secrete VEGF and TGF-β, while a parallel stress-induced neutrophilia and lymphopenia tilt the systemic neutrophil–lymphocyte and platelet–lymphocyte balance—together fostering an immunosuppressive milieu that helps residual tumor cells escape immune surveillance [6,7,8,9,10].

Three complete blood count (CBC)-derived indices capture this systemic response: the neutrophil-to-lymphocyte ratio (NLR), the platelet-to-lymphocyte ratio (PLR), and the systemic immune-inflammation index (SII), defined as platelet × neutrophil/lymphocyte. Each has been individually associated with EOC prognosis [9,10,11,12,13,14]. PLR captures the pro-tumorigenic contribution of platelets, which are active participants in the EOC tumor microenvironment through VEGF-mediated angiogenesis, TGF-β-driven immune evasion, and direct tumor cell stimulation [9,10]. NLR reflects the neutrophil–lymphocyte balance critical to anti-tumor immunity. SII integrates both dimensions, but is mathematically defined as platelet count (PLT) × NLR—a structural interrelationship with important implications for composite modeling.

Despite growing evidence for individual markers, two gaps remain. First, few studies have systematically compared perioperative ΔSII, ΔNLR, and ΔPLR within the same cohort across clinical subgroups to explore potential context-dependent differences in marker performance. Second, inflammatory markers alone provide modest discrimination: even the best single marker achieves an area under the curve (AUC) of approximately 0.60–0.65 for OS [11,12,13,14,15]. A related, practical question is how this perioperative inflammatory information might be combined with established surgical outcome variables—FIGO stage and residual disease—to support postoperative risk stratification.

Accordingly, the primary aim of this study was a systematic head-to-head comparison of perioperative ΔSII, ΔNLR, and ΔPLR across 10 pre-specified clinical subgroups, to characterize where and to what extent each marker is informative. Recognizing that a single marker is unlikely to be uniformly optimal across subgroups, we additionally explored—as a secondary, exploratory objective—whether integrating the inflammatory signal with FIGO stage and residual disease into a Clinical-Inflammatory Risk Score (CIRS) could refine postoperative risk stratification.

## 2. Results

### 2.1. Patient Characteristics

A total of 373 patients were included (Table 1). Median age was 53 years (interquartile range [IQR] 46–62). Advanced FIGO stage (II+) was present in 217 patients (58.2%), serous histology in 229 (61.4%), and high-grade tumors in 331 (88.7%). Complete cytoreduction (R0) was achieved in 303 patients (81.2%). Non-serous tumors had a notably lower recurrence rate (29.2%) than serous tumors (54.1%), reflecting earlier-stage presentation and more favorable disease biology [16,17]. Ninety-six deaths (25.7%) and 166 recurrences (44.5%) were recorded. Median follow-up was 53 months (IQR 43–59), estimated by the reverse Kaplan–Meier method.

### 2.2. Individual Marker Performance: Modest Standalone Discrimination

All three markers significantly stratified OS and PFS (Table 2, Figure S1). ΔPLR achieved the highest OS event-status AUC (0.615; 95% confidence interval [CI] 0.547–0.686), followed by ΔSII (0.604) and ΔNLR (0.593). For PFS, AUC ranged from 0.579 to 0.594. Importantly, all individual marker AUCs fell below 0.65, consistent with the modest standalone discrimination reported in prior studies [11,14]. No significant AUC differences were found between markers at the whole-cohort level (all pairwise DeLong *p* > 0.20), indicating that no single index demonstrated sufficient discrimination to support its use as a standalone prognostic marker. Censoring-aware metrics gave the same ordering: Harrell’s C-index for OS was 0.599 for ΔPLR, 0.570 for ΔSII and 0.559 for ΔNLR, and time-dependent AUCs at 1, 3 and 5 years showed the same pattern (Table S3).

### 2.3. Subgroup Analysis: Context-Dependent Performance

**Figure 1:**
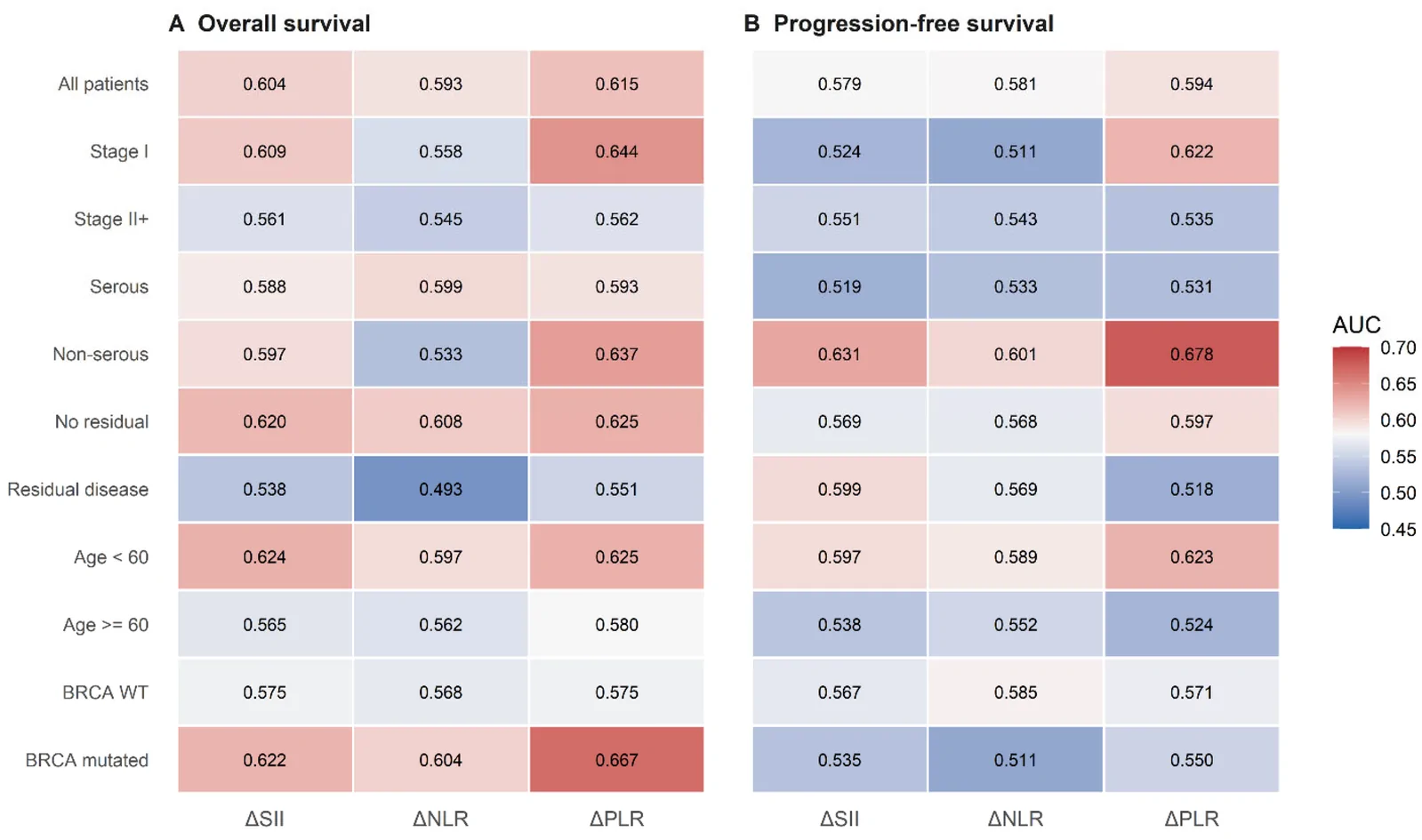
Subgroup event-status AUC heatmap for the full cohort and 10 pre-specified clinical subgroups (*N* = 373). (**A**) Overall survival; (**B**) progression-free survival. In both panels, rows represent the full cohort and the pre-specified subgroups, columns represent the three perioperative inflammatory indices (ΔSII, ΔNLR, ΔPLR), and cell values denote the corresponding AUC, with the color scale ranging from blue (lower discrimination) to red (higher discrimination). ΔPLR was the most discriminative marker in most subgroups for both OS and PFS; the best-performing marker and achievable discrimination varied by clinical context. Between-marker differences in small subgroups, including residual disease (*n* = 70), are exploratory. BRCA status was unknown or untested in 115 patients; therefore, the BRCA wild-type (*n* = 180) and BRCA-mutated (*n* = 78) subgroups do not sum to the full cohort. Confidence intervals for every subgroup AUC are provided in Table S4, and pairwise comparisons between markers in Table S5.

Although whole-cohort AUCs were similar across markers, the most discriminative marker and the level of discrimination achievable varied by clinical context (Table S1, Figure 1). For OS, ΔPLR showed the numerically highest AUC in the majority of subgroups, although several margins were narrow and ΔNLR was marginally higher in serous tumors. Discrimination was greater in lower-burden settings—Stage I (ΔPLR AUC 0.644 vs. 0.609 for ΔSII and 0.558 for ΔNLR), complete cytoreduction (0.625), and age < 60 (0.625)—and markedly reduced in higher-burden settings, with all three markers near chance in patients with residual disease (ΔPLR 0.551, ΔSII 0.538, ΔNLR 0.493). By histology, ΔPLR performed comparably in serous (0.593) and non-serous (0.637) tumors, whereas by molecular status its highest OS value occurred in BRCA-mutated tumors (0.667 vs. 0.575 in wild-type).

For PFS, the pattern differed. ΔPLR again led in most subgroups but with smaller margins; its PFS discrimination was weakest in serous tumors (0.531) and strongest in non-serous tumors, where it reached the highest value observed in any subgroup (0.678; ΔSII 0.631, ΔNLR 0.601). In a few small subgroups the numerical ranking differed—in patients with residual disease (*n* = 70) ΔSII showed the highest PFS value (0.599 vs. ΔPLR 0.518 and ΔNLR 0.569), and in BRCA-wild-type and older patients ΔNLR was marginally higher (BRCA-WT 0.585; age ≥ 60 0.552)—but these within-subgroup differences fall within exploratory margins and should not be over-interpreted.

Across subgroups, discrimination tracked tumor burden, being higher in lower-burden settings (early-stage, complete cytoreduction) and lower in residual or advanced disease; the platelet-containing indices (ΔPLR, ΔSII) generally showed the higher numerical values, with ΔPLR most often ranking first. These subgroup analyses rest on limited event counts (e.g., 13 OS events in Stage I and 36 in residual disease), so confidence intervals are wide and overlapping; the patterns are therefore exploratory and are interpreted mechanistically in the Section 3. Confidence intervals for all 66 subgroup AUC estimates are given in Table S4 and are wide (median width 0.176, maximum 0.342). In pairwise DeLong comparisons within subgroups (Table S5), only 3 of 44 tests reached nominal significance and none remained significant after Benjamini–Hochberg correction. The ranking of markers within subgroups is therefore descriptive and not statistically established.

### 2.4. CIRS Development and Internal Performance Evaluation

In ridge-penalized Cox regression, ΔSII carried a consistently negative coefficient (β = −0.78) across all methods and λ values—reflecting collinear suppression once ΔPLR and ΔNLR are jointly modeled—and was assigned zero weight; the resulting CIS = 0.465 × std(ΔNLR) + 0.535 × std(ΔPLR), where std(·) denotes standardization to zero mean and unit standard deviation. None of the five candidate clinical variables met the ΔAUC ≥ 0.01 threshold. The final CIRS comprised CIS (0–50 points), FIGO Stage II+ (30), and residual disease (20).

**Figure 2:**
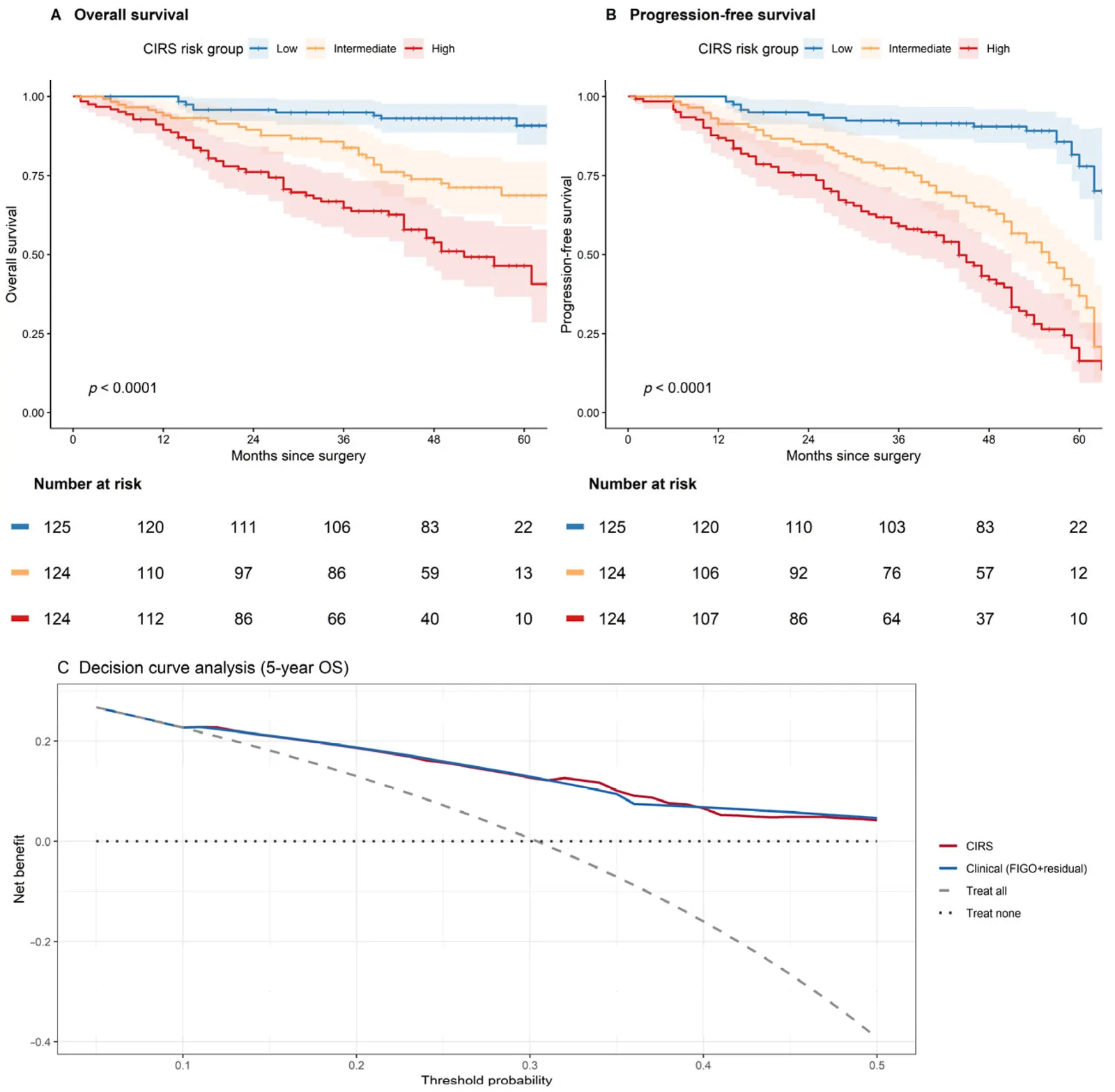
CIRS risk stratification and clinical utility (*N* = 373). (**A**) CIRS OS Kaplan–Meier: Low 7.2%, Intermediate 25.8%, High 44.4% mortality (~6-fold gradient; log-rank *p* < 0.001). (**B**) CIRS PFS Kaplan–Meier: Low 16.8%, Intermediate 47.6%, High 69.4% recurrence. Kaplan–Meier panels show 95% confidence bands with numbers at risk beneath each panel; follow-up is truncated at 60 months because fewer than five patients per group remained at risk thereafter. (**C**) Decision curve analysis (5-year OS): CIRS shows positive net benefit comparable to the clinical-only model across 10–40% threshold probability range.

CIRSs (range 0.0–81.4; median 32.5) defined three risk groups with a pronounced stepwise gradient (OS mortality 7.2%, 25.8%, 44.4%—approximately six-fold; PFS recurrence 16.8%, 47.6%, 69.4%; all log-rank *p* < 0.001; Table 3, Figure 2A,B). Discrimination increased from individual markers (AUC 0.579–0.615) and the clinical-only model (FIGO + residual; 0.732) to the full CIRS (0.752 OS, 0.764 PFS; Table S2, Figure S2A,B), with broadly consistent performance on internal validation (bootstrap-corrected C-index 0.726; five-fold cross-validation (CV) AUC 0.745 [OS]/0.757 [PFS]) and net benefit comparable to the clinical-only model on decision curve analysis (Figure 2C).

### 2.5. Multivariable Cox: Independent Contribution of Inflammatory Component

In a multivariable model adjusting for surgical complexity, estimated blood loss, age, histology, and anemia (available for 365 of 373 patients), the CIRS remained strongly associated with both OS (HR 2.55 per 1-SD increase, 95% CI 1.96–3.31, *p* < 0.001) and PFS (HR 2.24, 95% CI 1.81–2.78, *p* < 0.001), satisfying the proportional-hazards assumption (Schoenfeld *p* = 0.50 [OS], 0.93 [PFS]; Table 4, Figure S2C). Among the adjustment covariates, non-serous histology (OS HR 2.36, 95% CI 1.41–3.95, *p* = 0.001) and greater blood loss (OS HR 1.71, *p* = 0.029) were additionally associated with overall survival, whereas surgical complexity, age, and anemia were not independently prognostic. Decomposing the score, its inflammatory component (CIS) contributed independently to PFS (HR 1.21, 95% CI 1.06–1.38, *p* = 0.004) but not OS (HR 1.14, 95% CI 0.97–1.35, *p* = 0.11), indicating that the prognostic strength of the CIRS derives mainly from FIGO stage and residual disease, with an additional inflammatory contribution to progression risk.

### 2.6. Molecular Correlates of the Histotype-Dependent Marker Performance

**Figure 3:**
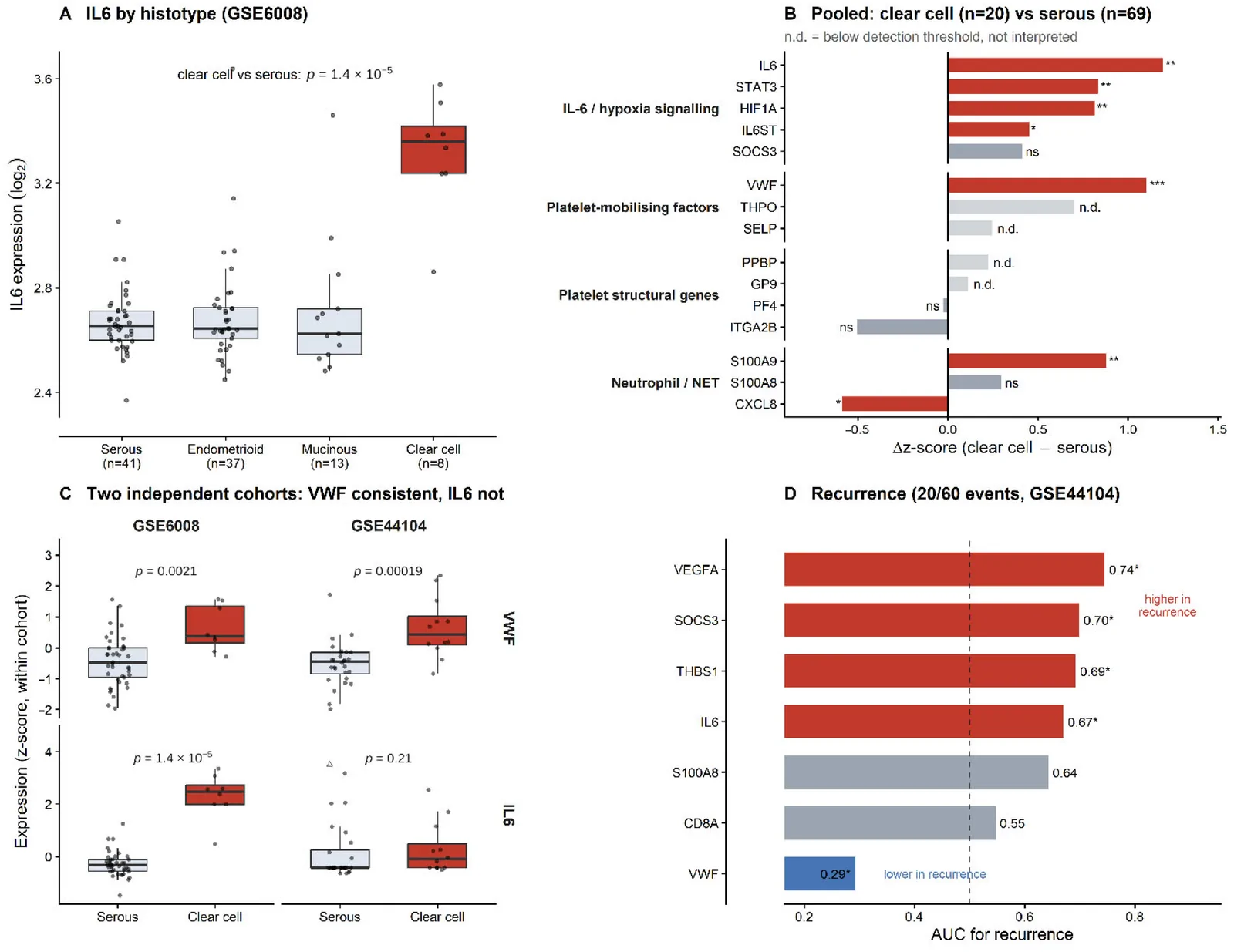
Molecular correlates of histotype-dependent marker performance in two independent public cohorts (GSE6008 and GSE44104). (**A**) IL6 expression by histotype in GSE6008; the elevation is confined to clear cell carcinoma. (**B**) Pooled comparison of clear cell (*n* = 20) versus serous (*n* = 69) carcinoma, grouped by biological role. Bars show Δ, the difference in within-cohort z-score between clear cell and serous carcinoma. Asterisks denote Benjamini–Hochberg FDR across the 15 genes shown (*** FDR < 0.001, ** FDR < 0.01, * FDR < 0.05); ns indicates FDR ≥ 0.05, and n.d. marks genes below the array detection threshold that were not interpreted. (**C**) The two cohorts presented in parallel: VWF is consistently higher in clear cell carcinoma, whereas the IL6 difference is present in GSE6008 only. The open triangle denotes a single value winsorized for display; all statistical tests used the untransformed data. (**D**) Exploratory discrimination of cumulative recurrence status in GSE44104 (20 of 60 patients); red denotes higher and blue lower expression in patients who recurred, with the dashed line at an AUC of 0.5. Here the asterisk denotes a nominal *p* < 0.05 for the corresponding AUC, from a Mann–Whitney U test comparing patients with and without recurrence and is not FDR-adjusted; it therefore carries a different meaning from the asterisks in (**B**). All *p* values are from Mann–Whitney U tests. These analyses provide molecular context for the clinical observations and are hypothesis-generating; they do not validate the perioperative biomarker findings.

Because the platelet-containing indices showed their highest numerical discrimination in non-serous tumors, two publicly available ovarian carcinoma transcriptomic datasets (GSE6008 [18] and GSE44104 [19]) were examined for a molecular counterpart (Figure 3). In GSE6008, IL6 expression was markedly higher in clear cell than in serous carcinoma (*p* = 1.4 × 10^−5^) but was not raised in endometrioid or mucinous tumors, indicating that the signal is confined to the clear cell histotype rather than shared across non-serous tumors as a group (Figure 3A). In an exploratory pooled z-score analysis (20 clear cell versus 69 serous), IL6, STAT3, HIF1A, IL6ST, VWF and S100A9 were higher and CXCL8 lower in clear cell carcinoma (all FDR < 0.05; Figure 3B). Treating the datasets as parallel independent cohorts, VWF was the only gene consistent in both (*p* = 0.0021 and *p* = 0.00019), whereas the IL6 difference observed in GSE6008 was not reproduced in GSE44104 (*p* = 0.21), even though the latter contained more clear cell tumors (12 versus 8) (Figure 3C). Platelet-specific transcripts either fell below the detection threshold of the arrays in tumor tissue (THPO, SELP, PPBP, GP9) or did not differ between histotypes (PF4, ITGA2B). In GSE44104, higher VEGFA, SOCS3, THBS1 and IL6 expression was associated with recurrence (AUC 0.67–0.74), whereas VWF showed the opposite direction (AUC 0.29; Figure 3D), indicating that its histotype association should not be read as a marker of adverse prognosis.

## 3. Discussion

Perioperative CBC-derived inflammatory indices provide a non-invasive, dynamic readout of the systemic immune-inflammatory response elicited by cytoreductive surgery and, by extension, a peripheral surrogate of the evolving tumor microenvironment. In this cohort, perioperative inflammatory markers provided only modest whole-cohort discrimination, with no significant difference among ΔSII, ΔNLR, and ΔPLR. We additionally examined performance across pre-specified subgroups and observed that the most discriminative marker differed by clinical context, and that discrimination tended to be higher in lower-burden disease. These are descriptive, hypothesis-generating observations; nonetheless, they are internally consistent and may help explain why single markers have shown variable effect sizes across heterogeneous cohorts. As a secondary exploratory analysis, we also developed a composite score (CIRS) integrating the inflammatory signal with established clinical and surgical prognostic factors; it showed modestly higher discrimination than the individual indices, which we regard as a secondary, exploratory result.

Although exploratory, these subgroup patterns are biologically plausible. First, tumor burden may modulate the dynamic range of the perioperative signal: because the Δ-markers isolate the surgical inflammatory response, they may be most informative when surgery is the dominant stimulus (stage I, complete cytoreduction), whereas pre-existing tumor-driven inflammation in bulky or residual disease [8] may compress the perioperative change. Second, the platelet-containing indices (ΔPLR, ΔSII) performed relatively better in non-serous tumors (ΔPLR PFS AUC 0.678); clear cell carcinoma in particular is characterized by constitutive interleukin-6 overexpression, which concurrently drives paraneoplastic thrombocytosis and STAT3-mediated tumor progression, amplifying platelet–tumor crosstalk and hence the biological signal captured by the platelet-containing indices [9,10]; endometrioid tumors share this interleukin-6-driven platelet biology to a lesser degree, whereas in high-grade serous tumors, stage and residual disease dominate the prognostic landscape, leaving little discriminative room (serous PFS AUC 0.52–0.53). Third, the numerically higher PFS AUC of ΔSII in patients with residual disease may be consistent with macroscopic tumor simultaneously driving platelet activation and neutrophil recruitment, the combined axis captured by SII’s multiplicative structure. Fourth, molecular context may further modulate marker performance: BRCA-mutated (homologous recombination-deficient) tumors carry a higher neoantigen load and a more T-cell-inflamed microenvironment enriched for tumor-infiltrating lymphocytes [20], so the lymphocyte term shared by all three indices reflects a qualitatively different, more actively engaged immune compartment than in homologous recombination-proficient tumors. Consistent with this altered immune contexture, ΔPLR was most discriminative for OS in BRCA-mutated tumors, whereas a neutrophil-weighted signal (ΔNLR) was marginally favored in wild-type and older patients. These estimates rest on limited events (e.g., 13 OS events in stage I), so individual differences may be unstable; only the burden-dependent trend was consistent across axes.

The consistent negative coefficient for ΔSII across all methods reflects collinear suppression: SII = PLT × NLR means that once ΔPLR and ΔNLR are jointly modeled, SII adds no independent information at the whole-cohort level. This finding likely reflects structural redundancy rather than a lack of prognostic relevance of ΔSII, which still showed context-dependent performance in the subgroup analyses. In plain terms, the zero weight does not mean ΔSII is clinically meaningless; because SII is by definition the product of platelet count and NLR, it adds little information once ΔNLR and ΔPLR are already in the model, even though it retained independent value in specific contexts such as residual disease. The multi-method sensitivity analysis (Ridge, LASSO, Elastic Net, across all λ values) consistently assigned β < 0 to ΔSII, providing methodological transparency regarding the assigned weights.

Residual disease and FIGO stage dominate prognostic stratification (clinical-only AUC 0.732), and the CIRS adds +0.020 (OS) and +0.011 (PFS) over this baseline. While these increments do not reach DeLong significance (event-status AUC) versus the clinical-only model, DCA shows positive net benefit comparable to the clinical-only model across the 10–40% threshold range, and the multivariable analysis confirms an independent inflammatory contribution to progression-free survival after adjusting for surgical and clinical variables. The CIRS thus identifies a subset of patients who fare somewhat worse than their surgical variables alone would predict. Because the incremental AUC gain over the clinical-only model did not reach DeLong significance, the CIRS is best regarded as a modest refinement rather than a substantial improvement in discrimination; its most consistent descriptive feature is a stepwise ~six-fold OS mortality gradient across risk groups (7.2% vs. 44.4%). The censoring-aware metrics gave increments of the same order (C-index 0.723 versus 0.710 for OS and 0.717 versus 0.703 for PFS; Table S3). Taken together, the CIRS does not achieve a statistically significant improvement in discrimination over FIGO stage and residual disease, and its value should be understood as interpretable risk stratification rather than added discriminative power.

It is useful to place these indices alongside established prognostic factors and previously reported inflammatory markers. Our whole-cohort discrimination (event-status AUC 0.579–0.615; Harrell’s C-index 0.559–0.605 across OS and PFS) is closely comparable to that reported for baseline systemic inflammatory markers in apparent early-stage ovarian cancer [21], although that study assessed static preoperative values whereas the present analysis examined perioperative dynamic change. In both settings the discriminative capacity of inflammatory indices is modest. In this cohort, FIGO stage and residual disease alone reached an AUC of 0.732 for overall survival, substantially higher than any individual index, and CA-125, the established circulating marker in this disease, added no discriminative value once stage and residual disease were accounted for (Table S6). These comparisons indicate that perioperative inflammatory indices are best regarded as supplementary to, rather than competitive with, established prognostic factors.

The markedly lower recurrence of non-serous tumors (29.2% vs. 54.1%) reflects the distinct biology of endometrioid and clear cell subtypes, which present at earlier stages [17]. In the multivariable CIRS model, non-serous histology was independently associated with overall survival (HR 2.36, *p* = 0.001), though not with progression-free survival. Age ≥ 60 and anemia were not independently prognostic in the multivariable model.

These clinical observations have a plausible counterpart at the transcriptional level. Exploratory pooled analysis suggested enrichment of IL6–STAT3- and hypoxia-related signals in clear cell carcinoma relative to serous carcinoma. However, among the individual genes examined, only VWF was consistently elevated across both cohorts, whereas the IL6 difference was cohort-dependent. This pattern is compatible with, but does not independently confirm, the previously described inflammatory phenotype of clear cell carcinoma, in which the IL6–STAT3–HIF axis is characteristically overexpressed relative to serous carcinoma [22]. Notably, this signal was confined to clear cell tumors and was not a property of non-serous carcinoma as a group; the transcriptomic comparison matching our clinical dichotomy—all non-serous versus serous—showed no significant difference. Clear cell carcinoma nonetheless accounted for 57 of the 144 non-serous tumors in this cohort (40%), the largest single non-serous subtype (Table 1), making it plausible that this histotype contributed disproportionately to the performance of the platelet-containing indices in that subgroup. This remains an inference rather than a demonstration: our subgroup analyses were pre-specified at the serous versus non-serous level, and the indices were not evaluated within individual non-serous histotypes. Mechanistically, VWF is an endothelial and megakaryocyte product that mediates platelet adhesion along the endothelium [23], and therefore provides a tissue-level link between the tumor and the peripheral platelet compartment that the platelet-containing indices sample. Platelet-specific transcripts, by contrast, were either undetectable or unchanged in tumor tissue. The latter is expected rather than contradictory: platelets are blood-borne and are not represented in the tumor transcriptome, and thrombopoietin is produced principally by the liver, so tumor-tissue data cannot test the interleukin-6–thrombopoietin–thrombocytosis limb of the proposed cascade. These analyses are therefore hypothesis-generating and provide a molecular context for, rather than a validation of, the clinical findings.

A further consideration is that the systemic response to malignancy extends beyond the leukocyte and platelet compartments captured by the complete blood count. Iron metabolism represents a complementary axis: serum transferrin has been shown to carry prognostic information in ovarian cancer complicated by cancer-related functional iron deficiency [24]. The present study was restricted by design to indices derivable from the routine perioperative complete blood count, and iron-status parameters were not measured systematically in this retrospective cohort; hemoglobin-defined anemia was available and was evaluated as a candidate component of the CIRS but did not meet the inclusion criterion. The omission of iron-status markers is therefore a limitation of this work, and their prospective incorporation is a natural direction for refinement of the score.

Several limitations apply. First, this retrospective two-center study lacks external validation, and the marker cutoffs were derived by Youden’s index and the CIRS weights estimated in the same cohort used for survival analysis. Although bootstrap and cross-validation showed broadly consistent internal performance, these procedures assessed model performance while the binary cutoffs were obtained from the full dataset rather than re-derived within each resampling fold; the reported discrimination should therefore be regarded as optimistic and is likely to be lower on external validation. Second, markers were measured only at baseline and POD 1, which reflects acute surgical stress and may be affected by blood loss, transfusion, infection, or perioperative medications that were not fully adjusted, with no later timepoint available. Third, the subgroup analyses were exploratory and, in several strata, based on few events (e.g., 13 deaths in stage I, 27 in BRCA-mutated patients), so the context-dependent marker ranking is hypothesis-generating. Fourth, BRCA status was missing in ~31% of patients and homologous recombination deficiency (HRD) result was unavailable, and evolving adjuvant/maintenance therapy over 2016–2021 was not modeled, leaving residual confounding. The inflammatory component’s incremental AUC over the clinical-only model did not reach DeLong significance, so the value of the CIRS lies in integrated stratification rather than inflammatory discrimination per se. Fifth, discriminative performance was quantified by event-status AUC rather than time-dependent AUC or Harrell’s C-index; because this metric ignores censoring and follow-up duration, the reported AUCs—particularly small-subgroup and between-marker differences—should be read as exploratory relative comparisons rather than definitive rankings, and time-to-event conclusions rest on the Cox and log-rank analyses. Finally, the molecular correlates were derived from tumor-tissue transcriptomes of separate public cohorts rather than from the present patients, no peripheral blood or perioperative material was available for those datasets, and the clear cell subgroups were small (8 and 12 tumors); these analyses cannot establish a causal link between the tumor phenotype and the perioperative blood indices. Those subgroups were small because endometrioid carcinoma is the predominant non-serous subtype in the public cohorts, whereas clear cell carcinoma is the largest non-serous subtype in our own series; and because our clinical subgroup analyses were defined as serous versus non-serous, we could not test directly whether clear cell tumors account for the subgroup difference observed. In addition, the constants used to rescale the inflammatory composite to points are specific to this cohort, so the CIRS cannot be applied to an external population without either these constants or re-derivation of the rescaling.

## 4. Materials and Methods

### 4.1. Study Design and Patient Population

This multicenter retrospective cohort study enrolled consecutive patients undergoing primary cytoreductive surgery for EOC, fallopian tube cancer, or primary peritoneal cancer at Samsung Medical Center (SMC) and Kangbuk Samsung Hospital (KBSMC) between July 2016 and December 2021. Inclusion criteria: (1) pathologically confirmed diagnosis; (2) primary surgery as initial treatment; (3) CBC available at preoperative baseline and postoperative day 1 (POD 1). The study was approved by the IRBs of SMC (No. 2025-09-037) and KBSMC (No. 2025-08-073); informed consent was waived. This analysis is based on the same institutional cohort (SMC and KBSMC, July 2016–December 2021) as our prior study of perioperative ΔSII dynamics [25]; the analytic sample of the present study may differ slightly because it required complete data for ΔSII, ΔNLR, and ΔPLR, and the work is extended to a head-to-head subgroup comparison of the three indices and to development of the CIRS.

### 4.2. Perioperative Inflammatory Markers

NLR, PLR, and SII were calculated at preoperative baseline (within 30 days before surgery) and POD 1. Absolute neutrophil and lymphocyte counts were derived from white blood cell (WBC) × differential percentage/100; PLT was obtained directly from the CBC. Percentage change: Δmarker (%) = [(POD 1 − baseline)/baseline] × 100. Percentage change was used in preference to absolute POD 1 values to isolate the perioperative surgical inflammatory response from pre-existing tumor-associated inflammation. Youden-optimal binary cutoffs were derived by ROC analysis for OS.

### 4.3. Individual Marker and Subgroup Analysis

Each marker was evaluated by Kaplan–Meier analysis (log-rank) and univariable Cox regression. Discriminative performance was summarized as the area under the ROC curve (AUC) for cumulative overall-mortality and disease-recurrence status over the full follow-up period (binary event indicators; pROC package), with 1000-iteration bootstrap CIs. This event-status AUC does not incorporate censoring or time-to-event and is therefore reported as an exploratory comparative measure of discrimination; time-to-event inference rests on the Cox and log-rank analyses. As a sensitivity analysis, censoring-aware discrimination was additionally quantified by Harrell’s C-index and by inverse-probability-of-censoring-weighted time-dependent AUC at 1, 3 and 5 years (timeROC package); these results are reported in Table S3. Throughout, “prognostic” denotes association with outcome; no inference regarding prediction of response to a particular treatment is intended. Subgroup AUC analyses were pre-specified across 10 groups: FIGO stage (I vs. II+), histology (serous vs. non-serous), residual disease (none vs. any), age (<60 vs. ≥60), and BRCA status (wild-type vs. mutated). These axes were pre-specified because each represents an established source of biological or clinical heterogeneity in EOC: FIGO stage and residual disease define tumor burden, the dominant prognostic determinants and plausible modifiers of the perioperative inflammatory signal; histology separates serous from non-serous tumors, which differ in platelet and inflammatory biology; age reflects host immunosenescence; and BRCA status reflects differences in the tumor immune microenvironment and neoantigen load. Within each subgroup, the marker with the numerically highest AUC was compared with each of the other two by the DeLong test, and 95% confidence intervals for every subgroup AUC were obtained by the same method (Tables S4 and S5). No adjustment for multiplicity was applied to the pre-specified subgroup analyses themselves, which are descriptive; the 44 pairwise comparisons were adjusted by the Benjamini–Hochberg procedure.

### 4.4. CIRS Development

The CIRS was developed in three steps. First, ridge (L2-penalized) Cox regression was applied to standardized ΔSII, ΔNLR, and ΔPLR to derive weights for an inflammatory composite; ridge was chosen because SII is mathematically PLT × NLR, creating structural multicollinearity among the markers [26,27,28]. The penalty λ was selected by 5-fold cross-validation; weight estimates were stable across the penalty grid (λ = 0.001–1.0); LASSO and Elastic Net analyses yielded the same marker-retention pattern. Markers with negative coefficients were assigned zero weight, yielding the Composite Inflammatory Score (CIS). For an individual patient i, CIS_i = 0.465 × z(ΔNLR_i) + 0.535 × z(ΔPLR_i), where z(·) denotes standardization to zero mean and unit standard deviation within this cohort; the composite was then rescaled to points as CIS-points_i = 50 × (CIS_i − CIS_min)/(CIS_max − CIS_min), with CIS_min = −1.053 and CIS_max = 9.249, and the final score computed as CIRS_i = CIS-points_i + 30 × I(FIGO stage ≥ II) + 20 × I(residual disease present). Because CIS_min and CIS_max are cohort-specific constants, external application of the score requires either these constants or re-derivation of the rescaling in the new cohort. Second, five additional candidate variables (age ≥ 60, Charlson Comorbidity Index (CCI) ≥ 2, log CA-125, ascites, anemia [Hb < 12 g/dL]) were evaluated for inclusion, requiring an increase in AUC of at least 0.01 over the model already containing CIS, FIGO stage and residual disease, which is the relevant comparison when deciding whether to add a further component. A discrimination-based rule was chosen deliberately, because the objective was an interpretable additive point score in which each component contributes measurable discriminative value; the 0.01 threshold was pre-specified but pragmatic rather than derived from formal criteria. In response to peer review, the same five candidates were therefore re-examined using likelihood ratio tests and AIC, which led to the same conclusion (Table S6): none qualified for inclusion. Third, for exploratory risk stratification, the CIRS was empirically constructed on a 0–100 scale as CIS (0–50 points), FIGO stage II or higher (30 points), and residual disease (20 points), theoretical range 0–100 (observed range 0.0–81.4), with risk-group cut-offs at the tertiles of the observed distribution (5.3 and 35.6 points). Tertiles were chosen a priori because they yield risk groups of approximately equal size (125, 124 and 124 patients), which stabilizes the group-specific estimates, and because they avoid data-driven cut-point optimization, a recognized source of optimism. The point allocation was intended to provide an interpretable exploratory scoring framework rather than a definitive coefficient-derived clinical prediction model.

### 4.5. Multivariable Validation and Statistical Analysis

Standard Cox proportional hazards regression was used for PFS multivariable analysis; Firth’s penalized likelihood (coxphf package, R) was retained only as a separation sensitivity check, as the primary CIRS model did not enter residual disease as a separate covariate and therefore showed no complete separation [29,30]. CIS was standardized to unit SD before model entry; hazard ratios represent per-SD increases. For the primary multivariable model, the CIRS was entered as a continuous variable; because it incorporates FIGO stage and residual disease by construction, these two variables were not re-entered as covariates, and the score was evaluated against the remaining surgical and clinical factors (surgical complexity, estimated blood loss, age, histology, anemia) to assess its independent prognostic value. Surgical complexity was available for 365 of 373 patients, defining the analytic sample for the multivariable model. In a secondary decomposition, the inflammatory component (CIS) alone was entered in place of the full CIRS to isolate its independent contribution to OS and PFS. The proportional-hazards assumption was assessed by Schoenfeld residuals; it held for the CIRS, while covariates with time-varying effects (histologic subtype, anemia, surgical complexity) were addressed in stratified sensitivity analyses. Internal validation: 500-iteration bootstrap optimism-corrected C-index; 5-fold cross-validation AUC; decision curve analysis (DCA). All statistical analyses were performed in R version 4.5.3 (R Foundation for Statistical Computing, Vienna, Austria) using the following packages: survival (version 3.8-6) for Cox proportional hazards regression, survminer (version 0.5.2) for Kaplan–Meier analysis, pROC (version 1.19.0.1) for ROC analysis and DeLong comparisons, timeROC (version 0.4.1) for inverse-probability-of-censoring-weighted time-dependent AUC, glmnet (version 5.0) for penalized (ridge, LASSO and Elastic Net) Cox regression, coxphf (version 1.13.4) for Firth’s penalized likelihood, dcurves (version 0.5.1) for decision curve analysis, and ggplot2 (version 4.0.3) for graphics. Two-tailed *p* < 0.05 was considered statistically significant.

### 4.6. Public Transcriptomic Analysis

To place the subgroup-dependent behavior of the perioperative indices in a molecular context, two publicly available ovarian carcinoma datasets were retrieved from the Gene Expression Omnibus: GSE6008 [18] (99 carcinomas and 4 normal ovaries; 41 serous, 37 endometrioid, 13 mucinous and 8 clear cell; Affymetrix HG-U133A array, Affymetrix, Santa Clara, CA, USA) and GSE44104 [19] (60 carcinomas; 28 serous, 12 clear cell, 11 endometrioid and 9 mucinous, with recurrence and survival annotation; Affymetrix HG-U133 Plus 2.0 array). The two datasets were treated as parallel independent cohorts; findings are therefore reported as consistent or inconsistent between cohorts.

Probe identifiers were mapped to gene symbols using the official platform annotations (GPL96 and GPL570); probes matching more than one gene were discarded, and for genes represented by several probes the probe with the highest mean signal was retained. Expression values were z-transformed within each dataset before pooling. A gene was regarded as undetected, and excluded from interpretation, when its median expression fell below the tenth percentile of the array and more than half of the samples shared the array floor value; on this criterion, THPO, SELP, PPBP and GP9 were undetectable in GSE44104. Clear cell and serous carcinomas were compared by Mann–Whitney U test with Benjamini–Hochberg correction applied across the genes displayed in each panel, and association with recurrence was summarized by the area under the ROC curve. Recurrence AUCs were descriptive and did not account for follow-up duration or censoring. All analyses were implemented independently in Python version 3.13 (Python Software Foundation, Wilmington, DE, USA) using NumPy, pandas, SciPy and scikit-learn, and in R version 4.5.3; the two implementations agreed to four significant figures.

## 5. Conclusions

In this two-center cohort of 373 patients undergoing primary cytoreductive surgery for epithelial ovarian cancer, perioperative changes in complete blood count-derived inflammatory indices carried only modest prognostic information, and no single index was statistically superior to the others, either in the cohort as a whole or within any subgroup. Integrating this inflammatory signal with FIGO stage and residual disease in the exploratory CIRS produced a score that was independently associated with overall and progression-free survival and that separated patients into groups spanning an approximately six-fold difference in mortality. Its discrimination, however, was not significantly better than that of stage and residual disease alone, and the prognostic strength of the score derived chiefly from those two established factors. Re-analysis of public transcriptomic data placed these observations in a molecular context and identified von Willebrand factor as consistently higher in clear cell than in serous carcinoma, but could not establish a causal link to the perioperative blood indices. The CIRS should therefore be regarded as an exploratory framework for integrated postoperative risk stratification rather than a validated clinical tool. Prospective external validation, evaluation of complementary biomarker axes such as iron metabolism, and assessment within individual histological subtypes are the necessary next steps.

## Figures and Tables

**Table 1:** Baseline clinical characteristics (*N* = 373).

Characteristic	Value
**Continuous variables**	
Age, years, median [IQR]	53 [46–62]
CA-125, U/mL, median [IQR]	252 [42–809]
**FIGO Stage**	
Stage I	156 (41.8%)
Stage II+	217 (58.2%)
**Histological subtype**	
Serous	229 (61.4%)
Non-serous	144 (38.6%)
Clear cell	57 (15.3%)
Mucinous	43 (11.5%)
Endometrioid	22 (5.9%)
Mixed	10 (2.7%)
Seromucinous	4 (1.1%)
Other	8 (2.1%)
**Grade**	
Grade 1	42 (11.3%)
Grade 2–3	331 (88.7%)
**Residual disease**	
R0 (no gross residual)	303 (81.2%)
R1-2 (any residual)	70 (18.8%)
**BRCA status**	
Wild-type	180 (48.3%)
Mutated	78 (20.9%)
Unknown/not tested	115 (30.8%)
**Other**	
Surgical complexity score (Aletti) †	
Low (≤3)	137 (37.5%)
Intermediate–high (≥4)	228 (62.5%)
Estimated blood loss, mL	400 (200–800)
Operation time, min	227 (169–290)
Ascites present	163 (43.7%)
CCI ≥ 2	153 (41.0%)
Deaths	96 (25.7%)
Recurrences	166 (44.5%)

CCI, Charlson Comorbidity Index. FIGO stage and tumor grade were dichotomized in the analytic dataset. † Available in 365 of 373 patients.

**Table 2:** Individual perioperative inflammatory marker performance (*N* = 373).

Marker	Cutoff (%Δ)	Fold Change	High-Risk n (%)	OS AUC	OS 95% CI	PFS AUC	PFS 95% CI	OS *p*	PFS *p*
ΔSII	≥99.0%	≥2.0×	229 (61.4%)	0.604	0.542–0.669	0.579	0.523–0.640	<0.001	0.043
ΔNLR	≥244.9%	≥3.4×	162 (43.4%)	0.593	0.527–0.653	0.581	0.515–0.633	0.001	<0.001
ΔPLR	≥14.8%	≥1.15×	240 (64.3%)	0.615	0.547–0.686	0.594	0.542–0.655	<0.001	<0.001

All AUC values < 0.65: modest standalone discrimination. Pairwise marker comparisons: all *p* > 0.20 (DeLong, event-status AUC; ns). Fold change = postoperative day-1 value relative to the preoperative baseline (1 + %Δ/100); cutoffs are the Youden-optimal %Δ thresholds for OS.

**Table 3:** CIRS risk group outcomes and discriminative performance (*N* = 373).

Risk Group	*N*	CIRS pts	Deaths *n* (%)	Recur. *n* (%)	OS 1/3/5yr	PFS 1/3yr	OS *p* vs. Low	PFS *p* vs. Low
Low	125	≤5.3	9 (7.2%)	21 (16.8%)	100%/95%/91%	100%/91%	Reference	Reference
Intermediate	124	5.3–35.6	32 (25.8%)	59 (47.6%)	94%/84%/69%	91%/77%	<0.001	<0.001
High	124	>35.6	55 (44.4%)	86 (69.4%)	89%/65%/46%	87%/59%	<0.001	<0.001

CIRS cutoffs: 33rd percentile = 5.3; 67th percentile = 35.6. ~6-fold OS mortality gradient (7.2% vs. 44.4%). CIRS AUC: OS = 0.752 (0.697–0.803); PFS = 0.764 (0.715–0.815). Bootstrap-corrected C-index = 0.726 (optimism = 0.000). 5-fold CV AUC: OS = 0.745 ± 0.012; PFS = 0.757 ± 0.006.

**Table 4:** Multivariable Cox regression of the Clinical-Inflammatory Risk Score (CIRS, continuous) adjusted for surgical and clinical covariates (*n* = 365).

Variable	OS HR (95% CI)	*p*	PFS HR (95% CI)	*p*
CIRS (per 1-SD increase)	2.55 (1.96–3.31)	<0.001	2.24 (1.81–2.78)	<0.001
Surgical complexity score ≥ 4	0.92 (0.58–1.46)	0.713	1.37 (0.92–2.02)	0.119
Estimated blood loss ≥ 500 mL	1.71 (1.06–2.77)	0.029	1.33 (0.93–1.91)	0.114
Age ≥ 60	1.00 (0.64–1.55)	0.988	0.95 (0.67–1.34)	0.771
Histology (non-serous)	2.36 (1.41–3.95)	0.001	1.36 (0.88–2.08)	0.163
Anemia	1.15 (0.75–1.76)	0.532	1.11 (0.80–1.54)	0.525

Standard Cox proportional-hazards regression (365 of 373 patients with complete surgical data). The CIRS was modeled as a continuous variable (per 1-SD increase); because it incorporates FIGO stage and residual disease by construction, these variables were not re-entered as covariates and the score was adjusted for the remaining surgical and clinical factors. The proportional-hazards assumption was satisfied (Schoenfeld *p* = 0.50 [OS], 0.93 [PFS]). Decomposing the score, the inflammatory component (CIS, per 1-SD increase) contributed independently to PFS (HR 1.21, 95% CI 1.06–1.38, *p* = 0.004) but not OS (HR 1.14, 95% CI 0.97–1.35, *p* = 0.11).

## Data Availability

The data presented in this study are available on request from the corresponding author. The data are not publicly available owing to privacy and ethical restrictions. The public transcriptomic datasets re-analyzed in this study are available from the Gene Expression Omnibus under accessions GSE6008 and GSE44104.

## Supplementary Materials

The following supporting information can be downloaded at: https://www.mdpi.com/article/10.3390/ijms27177777/s1.
