# Supplementary material for: Systemic Immune-Inflammatory Biomarkers in Epithelial Ovarian Cancer: Subgroup-Dependent Prognostic Performance and Integrated Risk Stratification
Source: Int J Mol Sci. 2026 Aug 30;27(17):7777. doi: 10.3390/ijms27177777 (PMC13566507; doi:10.3390/ijms27177777)
Supplement: Supplementary file 1 [file ijms-27-07777-s001.zip › ijms-4489312-supplementary.pdf]

-Supplementary Materials

Table S1. Subgroup AUC Analysis: ΔSII vs. ΔNLR vs. ΔPLR (*N* = 373)

Subgroup	*N*	ΔSII OS	ΔNLR OS	ΔPLR OS	ΔSII PFS	ΔNLR PFS	ΔPLR PFS
All patients	373	0.604	0.593	0.615	0.579	0.581	0.594
Stage I	156	0.609	0.558	0.644	0.524	0.511	0.622
Stage II+	217	0.561	0.545	0.562	0.551	0.543	0.535
Serous	229	0.588	0.599	0.593	0.519	0.533	0.531
Non-serous	144	0.597	0.533	0.637	0.631	0.601	0.678
No residual	303	0.620	0.608	0.625	0.569	0.568	0.597
Residual disease	70	0.538	0.493	0.551	0.599	0.569	0.518
Age < 60	253	0.624	0.597	0.625	0.597	0.589	0.623
Age ≥ 60	120	0.565	0.562	0.580	0.538	0.552	0.524
BRCA WT	180	0.575	0.568	0.575	0.567	0.585	0.571
BRCA mutated	78	0.622	0.604	0.667	0.535	0.511	0.550

Table S2. Model AUC Comparison (*N* = 373)

Model	OS AUC	OS 95% CI	PFS AUC	PFS 95% CI
Best individual marker (ΔPLR)	0.615	0.547–0.686	0.594	0.542–0.655
Clinical only (FIGO + Residual)	0.732	0.680–0.783	0.753	0.706–0.799
CIRS (Inflammatory + FIGO + Residual)	0.752	0.697–0.803	0.764	0.715–0.815

DeLong: CIRS vs. ΔPLR p<0.01; CIRS vs. Clinical only OS p=0.22; PFS p=0.42.

Table S3. Time-dependent AUC (1, 3 and 5 years) and Harrell's C-index (*N* = 373)

Predictor	OS 1y	OS 3y	OS 5y	OS C-index	PFS 1y	PFS 3y	PFS 5y	PFS C-index
ΔSII	0.461	0.568	0.639	0.570	0.540	0.590	0.626	0.570
ΔNLR	0.463	0.557	0.611	0.559	0.542	0.578	0.608	0.567
ΔPLR	0.537	0.618	0.648	0.599	0.672	0.655	0.640	0.605
CIS	0.503	0.607	0.640	0.592	0.634	0.639	0.634	0.602
Clinical-only	0.773	0.746	0.736	0.710	0.757	0.707	0.716	0.703
CIRS	0.754	0.762	0.753	0.723	0.771	0.735	0.746	0.717

Events at 1 / 3 / 5 years: OS 20 / 65 / 93; PFS 26 / 83 / 152. One-year estimates rest on few events. The CIRS exceeds the clinical-only model at 3 and 5 years for both endpoints and by C-index; at 1 year for OS the clinical-only model is numerically higher (0.773 vs. 0.754), consistent with the small number of early deaths. ΔPLR remains the best-performing individual marker at every landmark, reproducing the event-status AUC ordering.

Table S4. Subgroup event-status AUC with DeLong 95% confidence intervals

Subgroup	*N*	Events	Outcome	Marker	AUC	95% CI	Width
All patients	373	96	OS	ΔSII	0.604	0.540–0.668	0.128
All patients	373	96	OS	ΔNLR	0.593	0.528–0.659	0.131
All patients	373	96	OS	ΔPLR	0.615	0.552–0.678	0.127
All patients	373	166	PFS	ΔSII	0.579	0.520–0.637	0.117
All patients	373	166	PFS	ΔNLR	0.581	0.523–0.640	0.118
All patients	373	166	PFS	ΔPLR	0.594	0.535–0.652	0.117
Stage I	156	13	OS	ΔSII	0.609	0.446–0.772	0.326
Stage I	156	13	OS	ΔNLR	0.558	0.396–0.721	0.324
Stage I	156	13	OS	ΔPLR	0.644	0.501–0.788	0.287
Stage I	156	27	PFS	ΔSII	0.524	0.406–0.642	0.236
Stage I	156	27	PFS	ΔNLR	0.511	0.394–0.628	0.233
Stage I	156	27	PFS	ΔPLR	0.622	0.514–0.731	0.218
Stage II+	217	83	OS	ΔSII	0.561	0.484–0.638	0.154
Stage II+	217	83	OS	ΔNLR	0.545	0.467–0.623	0.156
Stage II+	217	83	OS	ΔPLR	0.562	0.485–0.639	0.154
Stage II+	217	139	PFS	ΔSII	0.551	0.470–0.632	0.162
Stage II+	217	139	PFS	ΔNLR	0.543	0.464–0.622	0.158
Stage II+	217	139	PFS	ΔPLR	0.535	0.455–0.615	0.160
Serous	229	65	OS	ΔSII	0.588	0.510–0.667	0.157
Serous	229	65	OS	ΔNLR	0.599	0.521–0.677	0.157
Serous	229	65	OS	ΔPLR	0.593	0.515–0.672	0.157
Serous	229	124	PFS	ΔSII	0.519	0.443–0.594	0.151
Serous	229	124	PFS	ΔNLR	0.533	0.458–0.608	0.150
Serous	229	124	PFS	ΔPLR	0.531	0.456–0.606	0.150
Non-serous	144	31	OS	ΔSII	0.597	0.477–0.717	0.240
Non-serous	144	31	OS	ΔNLR	0.533	0.410–0.657	0.247
Non-serous	144	31	OS	ΔPLR	0.637	0.525–0.749	0.225
Non-serous	144	42	PFS	ΔSII	0.631	0.528–0.733	0.205
Non-serous	144	42	PFS	ΔNLR	0.601	0.497–0.705	0.208
Non-serous	144	42	PFS	ΔPLR	0.678	0.583–0.773	0.190
No residual	303	60	OS	ΔSII	0.620	0.541–0.699	0.158
No residual	303	60	OS	ΔNLR	0.608	0.529–0.687	0.158
No residual	303	60	OS	ΔPLR	0.625	0.549–0.701	0.152
No residual	303	116	PFS	ΔSII	0.569	0.500–0.637	0.137
No residual	303	116	PFS	ΔNLR	0.568	0.500–0.636	0.136
No residual	303	116	PFS	ΔPLR	0.597	0.530–0.664	0.134
Residual disease	70	36	OS	ΔSII	0.538	0.398–0.679	0.281
Residual disease	70	36	OS	ΔNLR	0.493	0.355–0.630	0.275
Residual disease	70	36	OS	ΔPLR	0.551	0.412–0.689	0.277
Residual disease	70	50	PFS	ΔSII	0.599	0.428–0.770	0.342
Residual disease	70	50	PFS	ΔNLR	0.569	0.416–0.722	0.307
Residual disease	70	50	PFS	ΔPLR	0.518	0.353–0.683	0.329
Age < 60	253	58	OS	ΔSII	0.624	0.538–0.709	0.171
Age < 60	253	58	OS	ΔNLR	0.597	0.509–0.684	0.176

Subgroup	*N*	Events	Outcome	Marker	AUC	95% CI	Width
Age < 60	253	58	OS	ΔPLR	0.625	0.541–0.708	0.167
Age < 60	253	103	PFS	ΔSII	0.597	0.525–0.669	0.144
Age < 60	253	103	PFS	ΔNLR	0.589	0.516–0.662	0.147
Age < 60	253	103	PFS	ΔPLR	0.623	0.552–0.694	0.142
Age ≥ 60	120	38	OS	ΔSII	0.565	0.461–0.669	0.207
Age ≥ 60	120	38	OS	ΔNLR	0.562	0.456–0.668	0.212
Age ≥ 60	120	38	OS	ΔPLR	0.580	0.476–0.683	0.206
Age ≥ 60	120	63	PFS	ΔSII	0.538	0.434–0.642	0.208
Age ≥ 60	120	63	PFS	ΔNLR	0.552	0.449–0.656	0.207
Age ≥ 60	120	63	PFS	ΔPLR	0.524	0.419–0.628	0.209
BRCA WT	180	54	OS	ΔSII	0.575	0.487–0.664	0.177
BRCA WT	180	54	OS	ΔNLR	0.568	0.477–0.659	0.182
BRCA WT	180	54	OS	ΔPLR	0.575	0.486–0.664	0.178
BRCA WT	180	91	PFS	ΔSII	0.567	0.483–0.651	0.168
BRCA WT	180	91	PFS	ΔNLR	0.585	0.501–0.668	0.167
BRCA WT	180	91	PFS	ΔPLR	0.571	0.487–0.655	0.169
BRCA mutated	78	27	OS	ΔSII	0.622	0.489–0.754	0.265
BRCA mutated	78	27	OS	ΔNLR	0.604	0.470–0.738	0.268
BRCA mutated	78	27	OS	ΔPLR	0.667	0.538–0.796	0.258
BRCA mutated	78	51	PFS	ΔSII	0.535	0.403–0.668	0.265
BRCA mutated	78	51	PFS	ΔNLR	0.511	0.380–0.642	0.262
BRCA mutated	78	51	PFS	ΔPLR	0.550	0.419–0.681	0.262

Confidence interval width: median 0.176, maximum 0.342. Wide intervals reflect small event counts in several strata.

Table S5. Pairwise DeLong comparisons of markers within subgroups, Benjamini–Hochberg adjusted

Subgroup	Outcome	Comparison	AUC1	AUC2	ΔAUC	*p*	*FDR*
All patients	OS	ΔPLR vs ΔSII	0.615	0.604	0.011	0.622	0.873
All patients	OS	ΔPLR vs ΔNLR	0.615	0.593	0.022	0.441	0.873
All patients	PFS	ΔPLR vs ΔSII	0.594	0.579	0.015	0.453	0.873
All patients	PFS	ΔPLR vs ΔNLR	0.594	0.581	0.012	0.614	0.873
Stage I	OS	ΔPLR vs ΔSII	0.644	0.609	0.036	0.542	0.873
Stage I	OS	ΔPLR vs ΔNLR	0.644	0.558	0.086	0.190	0.873
Stage I	PFS	ΔPLR vs ΔSII	0.622	0.524	0.099	0.023	0.493
Stage I	PFS	ΔPLR vs ΔNLR	0.622	0.511	0.111	0.034	0.493
Stage II+	OS	ΔPLR vs ΔSII	0.562	0.561	0.001	0.977	0.985
Stage II+	OS	ΔPLR vs ΔNLR	0.562	0.545	0.017	0.595	0.873
Stage II+	PFS	ΔSII vs ΔNLR	0.551	0.543	0.008	0.635	0.873
Stage II+	PFS	ΔSII vs ΔPLR	0.551	0.535	0.016	0.502	0.873
Serous	OS	ΔNLR vs ΔSII	0.599	0.588	0.011	0.547	0.873
Serous	OS	ΔNLR vs ΔPLR	0.599	0.593	0.006	0.874	0.962
Serous	PFS	ΔNLR vs ΔSII	0.533	0.519	0.015	0.363	0.873
Serous	PFS	ΔNLR vs ΔPLR	0.533	0.531	0.002	0.950	0.985
Non-serous	OS	ΔPLR vs ΔSII	0.637	0.597	0.041	0.279	0.873
Non-serous	OS	ΔPLR vs ΔNLR	0.637	0.533	0.104	0.023	0.493
Non-serous	PFS	ΔPLR vs ΔSII	0.678	0.631	0.047	0.185	0.873
Non-serous	PFS	ΔPLR vs ΔNLR	0.678	0.601	0.077	0.081	0.832
No residual	OS	ΔPLR vs ΔSII	0.625	0.620	0.005	0.853	0.962
No residual	OS	ΔPLR vs ΔNLR	0.625	0.608	0.017	0.589	0.873
No residual	PFS	ΔPLR vs ΔSII	0.597	0.569	0.028	0.197	0.873
No residual	PFS	ΔPLR vs ΔNLR	0.597	0.568	0.029	0.263	0.873
Residual disease	OS	ΔPLR vs ΔSII	0.551	0.538	0.012	0.803	0.930
Residual disease	OS	ΔPLR vs ΔNLR	0.551	0.493	0.058	0.371	0.873
Residual disease	PFS	ΔSII vs ΔNLR	0.599	0.569	0.030	0.544	0.873
Residual disease	PFS	ΔSII vs ΔPLR	0.599	0.518	0.081	0.095	0.832
Age < 60	OS	ΔPLR vs ΔSII	0.625	0.624	0.001	0.963	0.985
Age < 60	OS	ΔPLR vs ΔNLR	0.625	0.597	0.028	0.402	0.873
Age < 60	PFS	ΔPLR vs ΔSII	0.623	0.597	0.026	0.259	0.873
Age < 60	PFS	ΔPLR vs ΔNLR	0.623	0.589	0.034	0.221	0.873
Age ≥ 60	OS	ΔPLR vs ΔSII	0.580	0.565	0.015	0.703	0.892
Age ≥ 60	OS	ΔPLR vs ΔNLR	0.580	0.562	0.017	0.730	0.892
Age ≥ 60	PFS	ΔNLR vs ΔSII	0.552	0.538	0.014	0.565	0.873
Age ≥ 60	PFS	ΔNLR vs ΔPLR	0.552	0.524	0.029	0.531	0.873
BRCA WT	OS	ΔSII vs ΔNLR	0.575	0.568	0.008	0.719	0.892
BRCA WT	OS	ΔSII vs ΔPLR	0.575	0.575	0.001	0.985	0.985
BRCA WT	PFS	ΔNLR vs ΔSII	0.585	0.567	0.018	0.353	0.873
BRCA WT	PFS	ΔNLR vs ΔPLR	0.585	0.571	0.014	0.692	0.892
BRCA mutated	OS	ΔPLR vs ΔSII	0.667	0.622	0.045	0.310	0.873
BRCA mutated	OS	ΔPLR vs ΔNLR	0.667	0.604	0.062	0.241	0.873
BRCA mutated	PFS	ΔPLR vs ΔSII	0.550	0.535	0.015	0.751	0.893
BRCA mutated	PFS	ΔPLR vs ΔNLR	0.550	0.511	0.038	0.460	0.873

3 of 44 comparisons reach nominal $p < 0.05$; none remains significant after Benjamini–Hochberg correction.

Table S6. Candidate variables re-evaluated over CIS + FIGO stage + residual disease (*N* = 373)

Candidate	Outcome	LRT χ^2	*p* (LRT)	ΔAIC	ΔAUC
Age ≥60	OS	0.037	0.8469	1.96	−0.004
CCI ≥2	OS	1.141	0.2855	0.86	0.000
log CA-125	OS	0.920	0.3376	1.08	−0.009
Ascites	OS	0.005	0.9431	1.99	−0.002
Anemia	OS	0.394	0.5303	1.61	−0.011
Age ≥60	PFS	0.024	0.8760	1.98	−0.002
CCI ≥2	PFS	0.418	0.5178	1.58	−0.008
log CA-125	PFS	1.675	0.1955	0.32	0.004
Ascites	PFS	4.157	0.0415	−2.16	0.003
Anemia	PFS	1.082	0.2982	0.92	−0.008

All five candidates fail the pre-specified ΔAUC ≥ 0.01 rule (range −0.011 to +0.004). Ascites reaches nominal LRT significance for PFS (*p* = 0.042, ΔAIC −2.16) but adds only +0.003 to discrimination; it is strongly prognostic univariably (HR 1.80 OS, 2.25 PFS) yet adds nothing once stage and residual disease are modelled, consistent with collinearity with tumor burden.

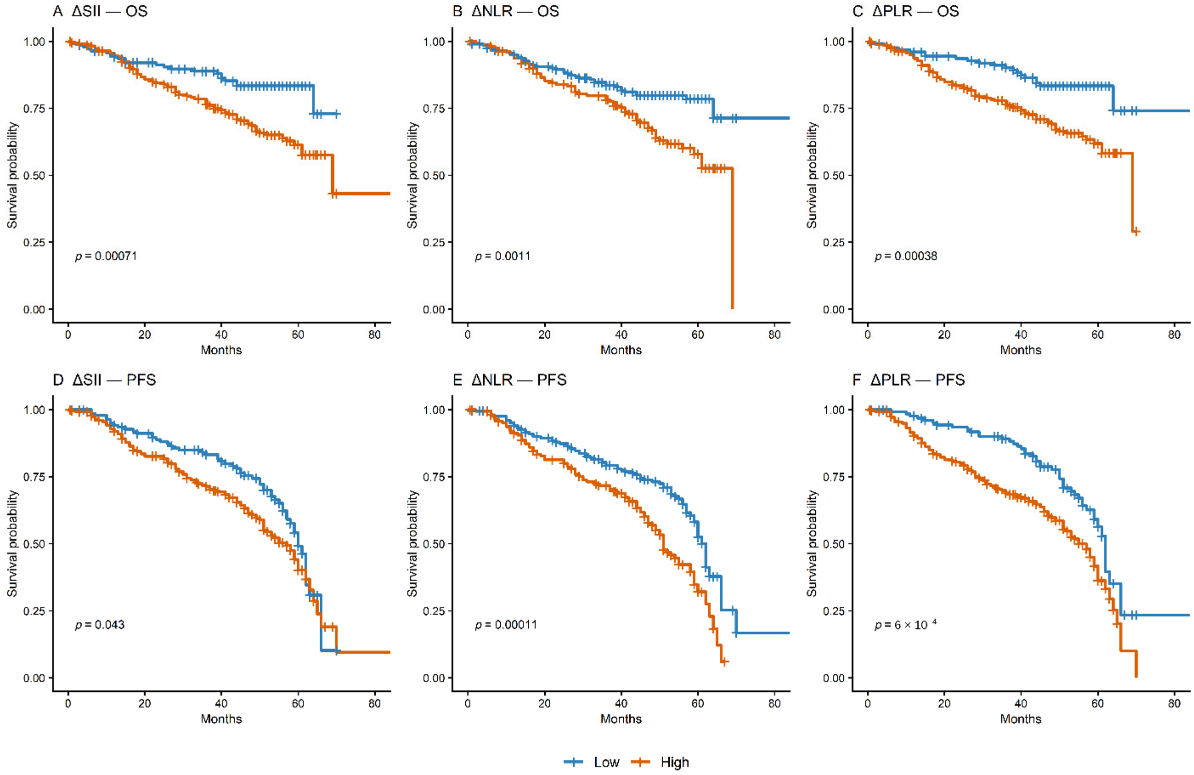

A ΔSII — OS
B ΔNLR — OS
C ΔPLR — OS
D ΔSII — PFS
E ΔNLR — PFS
F ΔPLR — PFS
Survival probability
Months
p = 0.00071
p = 0.0011
p = 0.00038
p = 0.043
p = 0.00011
p = 6 × 10⁻⁴
Low
High

Figure S1.

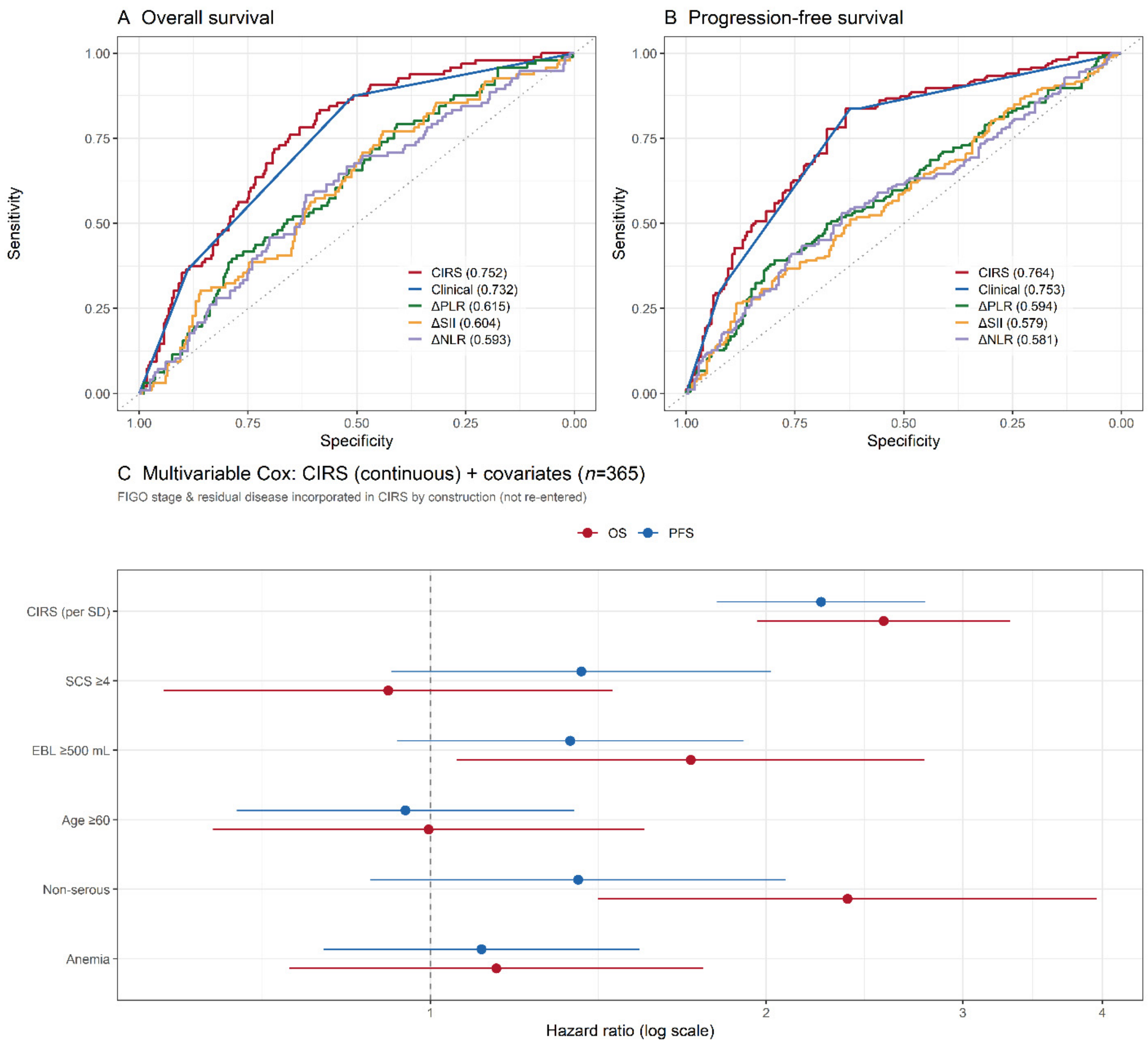

A Overall survival
B Progression-free survival
Sensitivity
Specificity
1.00
0.75
0.50
0.25
0.00
CIRS (0.752)
Clinical (0.732)
ΔPLR (0.615)
ΔSII (0.604)
ΔNLR (0.593)
CIRS (0.764)
Clinical (0.753)
ΔPLR (0.594)
ΔSII (0.579)
ΔNLR (0.581)
C Multivariable Cox: CIRS (continuous) + covariates (n=365)
FIGO stage & residual disease incorporated in CIRS by construction (not re-entered)
OS
PFS
CIRS (per SD)
SCS ≥4
EBL ≥500 mL
Age ≥60
Non-serous
Anemia
1
2
3
4
Hazard ratio (log scale)

Figure S2
